# Evidence-based policy lifecycle management: The H2020 DECIDO experience

**DOI:** 10.12688/openreseurope.15697.1

**Published:** 2023-05-02

**Authors:** Antonio Filograna, Fabio Perossini, Davide Prette

**Affiliations:** 1 Engineering Ingegneria Informatica S.p.A., Rome, Italy, Italy; 2 Key People Research Foundation, Valletta, Malta; 3 Volontariato Torino, Turin, Italy, Italy

**Keywords:** co-creation, policy life cycle, turin

## Abstract

Public administrations are an integral part of every state, and are currently changing to gradually improve weak policies in emergency management. Policies addressing emergencies such as floods, poverty and Ukrainian refugees are current issues in most European countries. Citizen engagement methodologies, data and digital technologies support this endeavour, yet the opportunities they offer are not still fully exploited in Europe. Furthermore, citizen perception of those policies could be more synchronised. In parallel, digital transformation, intensified by the exceptional circumstances of the coronavirus disease (COVID-19) crisis, is both imperative and inevitable for almost every aspect of the modern world and economy but a big issue remains to be addressed to boost the citizens’ participation in policy lifecycle development and in their evidencing real achievements. This creates both new needs and solutions for public administrations. This paper aims to identify the open needs encountered by public administrations, involving reflections on available methodologies, data, and tools towards evidence-based policy-making tailored to these needs. To accomplish that, knowledge, as represented by extensive desk research, was integrated with experience undertaken in the city of Turin, including the outcome of qualitative interviews with public administration experts. Key findings are discussed in detail concluding with a public administration needs ecosystem, mapping the needs, and uncovering similarities to support the replication of practices and procedures in different policymaking settings.

## Plain language summary

In this paper the DECIDO (eviDEnce and Cloud for more InformeD and effective pOlicies) project team will address how the project is facing three challenges related to policy management: 1) the policy lifecycle approach and its opportunities, 2) the supporting technologies to collect data and manage lifecycle processes provided by the European Open Science Cloud (EOSC) or developed from scratch, and 3) piloting of suggested solutions evaluating achieved results.

Co-creation emerged in the public sector literature related to service delivery around 10 years ago and was then transferred to public administration scholars. Public sector researchers broadened the term and adjusted it to fit the public sector, bringing in other theoretical perspectives, such as public administration and governance.

All over Europe the distance between citizens' needs and the political representation is a critical issue that needs to be addressed with the proper attention providing some innovative approach and tools. One of the main issues linked to policy co-creation is how to collect and manage data needed to formulate and relate to evidence. EOSC was identified as the preferred source of services and data.

During the last months coronavirus disease (COVID-19) pandemics and the Ukraine-Russia war evidenced solidarity unstructured self activated mechanisms in several areas involving citizens and associations in a full horizontal subsidiarity attitude. The Horizon 2020 DECIDO project started at the very beginning of the pandemic, and when it was written nobody had in mind the two arising emergencies we are now addressing. In particular this paper will concentrate on the need for a policy related to Ukrainian refugees in the city of Turin.

## Disclaimer

The EC's support for the production of this publication does not constitute an endorsement of the contents, which reflect the views only of the authors, and the Commission cannot be held responsible for any use which may be made of the information contained therein.

## Introduction

In this paper the DECIDO (eviDEnce and Cloud for more InformeD and effective pOlicies) project team will address how the project is facing three challenges related to policy management: 1) the policy lifecycle approach and its opportunities, 2) the supporting technologies to collect data and manage lifecycle processes provided by the European Open Science Cloud (EOSC) or developed from scratch, and 3) piloting of suggested solutions evaluating achieved results.

Co-creation emerged in the public sector literature related to service delivery around 10 years ago and was then transferred to public administration scholars. Public sector researchers broadened the term and adjusted it to fit the public sector, bringing in other theoretical perspectives, such as public administration and governance.

All over Europe the distance between citizens' needs and the political representation is a critical issue that needs to be addressed with the proper attention providing some innovative approach and tools ^1^.

One of main issues linked to policy co-creation is how to collect and manage data needed to formulate and relate to evidence. The EOSC was identified as the preferred source of services and data.

During the last months coronavirus disease (COVID-19) pandemics and the Ukraine-Russia war evidenced solidarity unstructured mechanisms that activated citizens in several areas in a full horizontal subsidiarity attitude.

H2020 DECIDO project started at the very beginning of the pandemic and when it was written nobody had in mind the two arising emergencies we are now addressing. In particular this paper will concentrate on the need for a policy related to Ukrainian refugees in the city of Turin.

## Methods

At the very beginning of the DECIDO project in Turin, COVID-19 co-creation initiatives benefited from strong engagement from co-creation partners who contributed resources including funding, time, staff, expertise, capacity and some basic technologies (Trello, Google Drive). This urgent purpose led to a common prioritisation of COVID-19 activities among all collaborators (even if other activities were slowed due to having lower priority). Providing social value was an important driver for many initiatives to support fragile citizens than suddenly included all the city population. More than 18,000 families were assisted in an incredible self-organised way. That was an ante-litteram co-creation experience.

**Figure 1.**
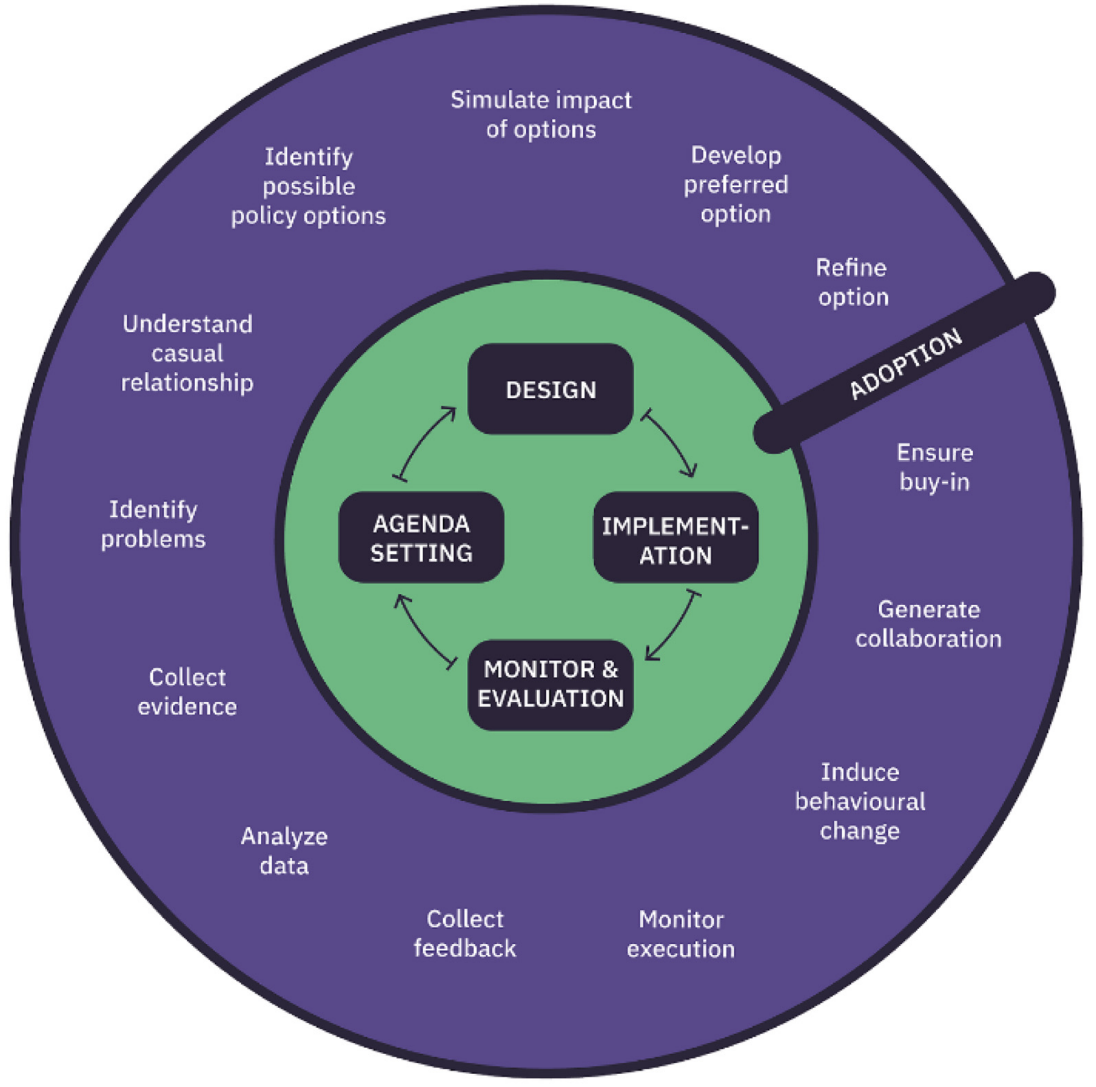
Policy life cycle.

We know that in other countries such as Estonia, Finland, Hungary and the UK, in various ways co-creation gave evidence of tremendous results even counting on poor resources. Recent events remind us how hard it is to foresee what the future may hold for citizens, communities, and their services. DECIDO, aiming at proposing the use of evidence-based policy life-cycle methodology ( Figure 1), is going to provide a feasible way to learn from evidence to build or improve the policy to better face citizens’ needs ^2^.

### Role of data

The potential of public administrations to generate data-driven evidence from modelling and simulations that complement or potentially replace traditional sources of evidence, such as scientific research and ex-post policy evaluation reports, has implications for the way these organisations function. Evidence-based policymaking is no longer reserved to scientists, statistical offices, and government evaluation departments but open to data scientists and public servants ^3^. In the DECIDO project, according to EU requirements, we emphasise the use of open public data collected by public administrations and civil society agencies. Data availability was considered a priority to give evidence the desired role in the project.

### The policy lifecycle methodology

The policy lifecycle concept is complex and not yet clearly defined, and it represents the latest in a series of efforts to enhance the efficiency and effectiveness of public policymaking through the application to policy formulation of an evaluative rationality. Evidence-based policymaking represents an effort to reform the process of policy formulation in such a way as to minimise non-design spaces. In that regard, evidence becomes the known relationships between policy instruments and policy goals, e.g. evaluation studies on the results or impacts of specific instruments, or the predictions of the impact of different instruments. The use of evidence in policymaking requires that policy actors have the analytical capacity to carry out such evaluation studies or predictions.

The methodology applied to DECIDO data plays a great role starting with the first phase, agenda setting. The challenge addressed is to detect (or even predict) problems before they become too costly to face, as well as reaching an agreement of which issues have to be dealt with. In this regard, through data, governments can identify emergent topics early and create relevant agenda points collecting data from social networks with high degrees of participation, and identifying citizens’ policy preferences.

The second phase, policy design, concerns the discussion related to the policy, its formation and acceptance, and the provision of means. Specifically, policy discussion deals with debating the different options on the table and identifying which is the most important. In this regard, opinion mining and sentiment analysis can help to inform policymakers about the current trend of the political discussion as well as the changes in public opinion in the light of discussed and proposed changes.

Policy formulation and acceptance deals with the use of big data and data analytics solutions for providing evidence for the ex-ante impact assessment of policy options, by helping to predict possible outcomes of the different options, by making use of advanced predictive analytics methodologies and scenario techniques. In that regard, robust and transparent predictive modelling and algorithmic techniques can also help in improving policy acceptance.

Finally, regarding provision of means, the challenge is to improve the decisions on how to provide the required personnel and financial means most efficiently for the implementation of new policies by analysing in detail past experiences. An example is the use of big data in budgeting to increase efficiency and effectiveness while reducing costs. In the third phase, implementation, big data, and data analytics can help identify the key stakeholders to be involved in policy or the key areas to be targeted by policies. Another way in which data can influence the implementation phase of the policy process is the real-time production of data. In fact, the execution of new policies immediately produces new data, which can be used to evaluate the effectiveness of policies and improve the future implementation processes ^4^.

### The policy lifecycle in DECIDO

Due to the short duration of the DECIDO project, the implementation phase is addressed through a simulation where the real implementation is not possible for regulatory, political, or financial issues. Finally, in the policy evaluation phase, the implementation of the policy provides quantitative and qualitative measures to assess performance and impact on users. Feedback and data on implementation can be collected and analysed by means of disruptive technologies. Clearly, predictions and simulations are deeply related to the agenda setting and policy design phases.

As simulation of a real policy decision is our priority, a clear concept will be implemented and prototyped for validation during the project lifecycle. The prototype is built and then validated by different stakeholders belonging to categories ranging from students to experts in specific domains. The prototype is built and validated so that the outcome of this activity is a final solution, well-working, validated and ready to be implemented after the needed ethical, engagement, technological and political steps:

Delivering and managing: launching the solution, i.e. delivering the solution to the stakeholders and managing its operation;

Evaluation and monitoring: tracking the performance of the solution to detect inefficiencies or possible improvements.

Iteration & improvement: co-creation is an iterative, circular process; as a result of the evaluation phase, it may be necessary to fix inefficiencies, implement improvements or even drop the solution and start the design process all over again.

Be prepared in emergencies: policy improvement has the potential to transform disaster risk management. Through four emergency policy pilots, the DECIDO project explores how policy impact prediction and analysis will lead to measurable outcomes and further policy refinement. Specifically, prediction is used mostly in the agenda setting and policy design phases of the policy cycle, in order to understand causal relationships and simulate the impact of policy options in view of their refinement. Specifically, there are four applications:

•   Pilot on forest fires in Kajaani (Finland). Policy objective: prevention and protection against forest fires; Procedures to mitigate damage to nature, infrastructure and life;

•   Pilots on floods in Meisino Park and in Murazzi area addressing residential, entrepreneurs and sportsters citizens - Turin (Italy). Policy objective: improve design of emergency policies related to floods and weather alerts;

•   Pilot on Social crises following the COVID-19 Pandemic and the Ukrainian refugees’ emergency after the outbreak of the war - Turin (Italy). Policy objective: define new policy to face specific social issues;

•   Pilot on power outages in Greek municipalities (Greece). Policy objective: power outage management of public infrastructure and cultural assets of Greek municipalities via emergency response mechanisms;

•   Pilot on wildfires in the Aragon Region (Spain). Policy objective: improve the design of emergency policies related to wildfires and management of controlled fires.

## Technologies

Policymaking is intended as a process to design, monitor and evaluate a policy ^5^. It is described as a set of activities to be undertaken to create a new policy or improve an existing one, involving the needed actors. These activities are part of the policy life cycle (PLC). There are several variations of the PLC, for example divided into three phases (planning, implementation, evaluation) or agenda setting, policy discussion, policy formulation and acceptance, provision of means, implementation ^6^. In the context of the DECIDO project, we decided to divide the PLC into four phases, namely agenda setting, policy formulation, policy implementation & simulation, policy evaluation, leveraging the cycle representation from Miles ^7^ and merging the phase of policy adoption and implementation to reduce the complexity without losing any information in the process.

For better specifying the activities to be undertaken in each of the four identified phases, each phase was divided into different steps according to the policy co-creation methodology designed in the DECIDO project.

The policymaking process consists of adoption of methodologies, data and digital technologies to support the steps in all the phases of the PLC. The offer of policy tools is various but at the same time fragmented, since a lot of single tools were provided for performing specific activities in the PLC. The success of a tool is related to a success of other tools ^8^. The policymakers do not have a comprehensive instrument to manage the policy creation process.

During the creation of a new policy, in order to facilitate the activities of the policymakers and all the actors involved in the PLC, a web portal was designed in the DECIDO project as a unique point of access to manage the whole policy creation and management process. The tool, named DECIDO Portal, is divided into two main areas, one public without any need to be registered where everybody can see the results of the co-creation activities developed during the PLC, and one private only for registered users to manage the activities and tools for creating a new policy. Before introducing the set of tools made available through the DECIDO Portal, a brief overview of the technologies used follows.

The portal aims at exploiting the European Cloud Infrastructure (ECI) through the direct collaboration with the EOSC, which provides storage capacity and processing power. We identified and integrated a set of tools and services that are useful for Public Authorities to take advantage of big data and cloud technologies, and to bring the citizen to the centre of the policy cycle. The DECIDO Portal provides public administrations with an easy-to-use interface to define, manage and evaluate their policies in a collaborative way by leveraging:

•   a distributed cloud platform available through the EGI (European Grid Infrastructure) Federated research cloud infrastructure which brings together tens of cloud providers across Europe. This cloud infrastructure allows DECIDO: 1) to run compute- or data-intensive tasks and host online services in virtual machines or docker containers on IT resources accessible via a uniform interface; 2) high-throughput data analysis to run compute-intensive tasks for producing and analysing large datasets and store/retrieve research data efficiently across multiple service providers; and 3) federated operations to manage services

•   data from other data providers (external to EOSC), including public administrations themselves (e.g. European Data Portal, ARPA Piemonte, OpenWeather).

•   other services available through EOSC, Catalogue and Marketplace (e.g. EGI Notebooks, Amnesia, EGI Online Storage, EGI Cloud Compute);

•   tools/services external to EOSC, for example tools already in use by public administrations and tools offered by the project partners in order to cover the whole evidence-based policy lifecycle

Even if the DECIDO will experiment its developed technological solution in the domain of disaster risk management (e.g. floods, wildfires, power outages), the DECIDO Portal aims to be domain agnostic.

The authentication and authorization system adopted by DECIDO Portal is EGI Check-in [11], an EOSC service provided by EGI. It was integrated with all the services offered by the portal. It also manages the roles used in the system, namely administrator, policy owner, member and visitor. If the user is registered and has the role as Administrator or Policy Owner, s/he can access to all the data used in the creation of the policy. The data are managed by another EOSC service, DataHub, which is an EGI component. This is directly integrated with the EGI Check-in as well as with other platform services such as: Jupyter Notebook, Data Analytics, Data Catalogue. DataHub can be used as the persistency layer, also allowing the possibility to share the data with other platform users.

The personal data are not managed by the DECIDO Portal. Before exploiting and sharing them, an anonymization process is needed. In the end, the users can analyse the aggregated data. To anonymize personal data, the DECIDO platform adopted Amnesia, an EOSC service provided by OpenAIRE. This tool allows to manage the personal data, anonymizing them. In order to leave the property of the personal data to public administrations, avoiding moving the data externally to their perimeter, an instance of Amnesia was installed in each municipality not accessible from outside. In this way, only authorised people can manage the data, anonymize them and finally provide them in an aggregated way.

One of the main strengths of the DECIDO Portal is that it integrates already existing tools which are to be used in different phases of the PLC, avoiding ‘reinventing the wheel’ and giving users the possibility to manage the entire policymaking process through a unique point of access. According to the needs and challenges resulting directly from the DECIDO pilot requirements, the portal integrates several tools to support all the actors involved in the PLC in their activities. Below, each tool will be briefly presented.

The DECIDO Survey Tool provides a user interface allowing, among other uses, the creation of customised surveys. This component will be widely used by the DECIDO stakeholders as it provides an easy way to construct complex surveys within a few minutes. From the other point of view, the survey tool facilitates citizens to provide their opinions or suggestions on specific matters.

The co-creation tool is a simple online tool that allows users to comment on each specific line of a document and allows everyone to see what comments were made by whom. It allows structured, open, many to many conversations.

For an efficient communication and collaboration between the different stakeholders it is crucial to establish a so called “single point of truth” for the specific knowledge in each policymaking project. The most important knowledge in DECIDO is obviously related to the key performance indicators (KPIs) to be used in defining a policy, the data used to support the KPIs and the policies as structured documents themselves. The policy editor component provides functionality to work on all project related assets like the gathering of data sources, definition of KPIs and the formulation of policies and helps to build and maintain the knowledge base.

The data catalogue is a component responsible for sharing open data published or collected by the DECIDO users. It provides a user with advanced functionalities to search for a dataset in DECIDO, see its description and download the data, or include it in the user's project.

The data analysis dashboard is a component that allows users to import data from various sources and create their own visualisations of the data to support policymaking. Data analysis tool enables advanced users, especially data analysts, to make deeper data analyses with Jupyter Notebook using for example Python algorithms or NLP (natural language processing) algorithms for extracting keywords from co-creation reports, or image analysis algorithms.

## The Turin pilot experience

The Turin pilot experience aims to improve the design of policies related to emergencies through the use of the co-creation methodology declined in all phases of the PLC and based on the use of the DECIDO Portal. In the submission phase of the DECIDO project (the beginning of 2020) the Turin pilot experience was designed to deal exclusively with the flood emergency; subsequently the new crises of COVID-19 and of the war between Russia and Ukraine led the project team to integrate these two factors of social emergencies into the project. The scenarios of the Turin pilot experience have therefore become the following:

1)   Flood emergency in two areas of the city of Turin, along the River Po: Murazzi and Meisino Park;

2)   Fighting against the food waste, in the increased distribution of basic necessities following the COVID-19 pandemic;

3)   Helping asylum seekers from Ukraine, to be welcomed in the best possible way after their arrival in Turin.

The co-creation sessions (held monthly) were divided into preparatory or restricted meetings, with more technical content, and into larger meetings, called “hackathons”, in which participants were invited to break down a macro-problem (e.g. the flooding emergency) into micro-problems to be tackled one at a time, bringing out reflections and possible solutions within the group with the help of a facilitator.

### Flood emergency

In the agenda setting phase, the participants from the two Turin areas involved in the co-creation process, i.e. inhabitants, entrepreneurs and managers of the local rowing clubs, highlighted since their first meeting many problems in the system of communicating weather warnings through e-mail and SMS by the local authorities: the analysis of the issued weather bulletins (collected in the data catalogue of the DECIDO Portal) showed poor comprehensibility of the messages, which made it difficult to react adequately and promptly to a flood alert.

As a consequence, the policy formulation phase led to the generation of many ideas about how to write and send effective messages regarding flood warnings: some ideas were abandoned at the selection and prioritisation stage, since they were not considered feasible, e.g. the proposal of developing an APP to receive tailored weather warnings, since it was considered very expensive and at risk of excluding portions of the population affected by the digital divide (e.g. the elderly). After some co-creation sessions and hackathons, together with the representatives of the Civil Protection Office of the City of Turin, the Piedmont Region, the Regional Environmental Protection Agency ARPA and the Piedmontese consortium for the automatic treatment of information - CSI, as well as of a local Psychologists’ association (Psicologi per i Popoli), the focus was put on the need for a website on which alert messages are collected and then sent automatically to different media with a linguistic register understandable to as many people as possible: so the policy implementation & simulation phase began with the categorisation of the messages to be collected and sent, taking into account the type of information, language and medium to be used depending on the potential recipient (e.g. youth or elderly, disabled or not, and so on).

### Fighting against food waste

A sharp increase in the demand for basic necessities following the COVID-19 pandemic has led to a simultaneous increase in the risk of food waste: many products that have already passed the "best before" expiration date indicated on the packaging are at risk of being thrown away because they are no longer considered consumable, even though they are still perfectly safe: a big communication and trust-building project is therefore needed. This was the main identified issue during the agenda setting phase of the PLC, by the volunteers of the local association CPD (Consulta per le Persone in Difficoltà), which runs a small warehouse for the collection and distribution of foodstuffs to people in need in Turin and in particular in a neighbourhood in the southern suburbs of the city: the products stored come from private donations or from the Piedmont branch of the Italian Food Bank.

As evidenced by the documents collected inside the data catalogue of the DECIDO Portal, there are already effective tools for communicating the recommended consumption interval, i.e. the period within which the product remains safe to be consumed despite having already passed its "best before" expiration date or Minimum Conservation Term (MCT), i.e. a comprehensive table presenting precisely the different recommended consumption intervals based on 16 product categories, approved by the Italian Ministry of Health. The problem is that it is not easy and quick to read.

Among the different ideas emerged during the policy formulation phase, one particularly stood out, given the immediacy of communication ensured by its strong visual impact: the creation of specific labels with a graphic design similar to those of the labels displaying information on the energy consumption of household appliances, in order to communicate, based on colours, how long each product can still be edible. In the policy implementation & simulation phase a printer capable of printing single labels was acquired. The need to contain purchasing costs led to the acquisition of a black-and-white machine, so the idea of printing labels in colour had to be abandoned. Currently under discussion is the type and amount of detail that the black and white label should contain: QR-code with a link to the above-mentioned table approved by the Italian Ministry of Health, "best before" expiration date, recommended consumption interval etc.

### Helping asylum seekers from Ukraine

The agenda setting phase related to the emergency scenario of refugees coming from Ukraine has brought to light a new type of emigration, mainly composed of women with children, who suddenly arrived all at once and who intend to stay in the host country - in this case Italy - for a tendentially short period. This implies a series of issues to be solved:

1) A difficulty of some institutions, which are not responding promptly to all requests for regularisation of residence documents or translation of certificates into Italian;

2) A lack of Ukrainian cultural mediators;

3) Even if, among Ukrainian refugees coming to Piedmont, there is an almost non-existent number of unaccompanied minors, there are many children with intellectual or mild motor disabilities, psychiatric or autistic disorders, together with elderly people with health problems that need to be better taken care of by the health services at a local level;

4) The data held by the Piedmont Region, the municipalities, the Civil Protection, the Prefectures and the local Ukrainian community do not coincide regarding the presence on the territory of Piedmont and Turin of Ukrainian refugees fleeing the war: this is most likely due to the fact that not all Ukrainian refugees arriving to Piedmont register with the police headquarters, as most of them are hosted by relatives already residing in Piedmont. If it is not possible to quantify the exact number of people in need, it becomes difficult to plan the actions to be implemented to concretely help Ukrainian refugees in the city of Turin and the surrounding area;

5) Ukrainian refugees are facing a lack of money: pensions, subsidies, bank accounts of many of them have been blocked (especially for those coming from cities that are at the moment under Russian control);

6) Refugees from Ukraine are often not given adequate psychological support.

The documentation uploaded on the data catalogue of the DECIDO Portal is very rich and includes the number of foreigners and asylum seekers in Italy, in the Piedmont Region and in the Metropolitan City of Turin, together with documentation concerning the historical presence of Ukrainian immigrants on the Piedmont territory, not as high as in other Italian regions, but still substantial even before the outbreak of the current war.

The team of stakeholders involved in the co-creation sessions is also very representative and includes members of the institutions (Piedmont Region, Municipality of Turin), large non-profit organisations at local level (e.g. the Youth Missionary Service SERMIG) and finally volunteers and small associations of the Ukrainian community in Turin.

During the policy formulation phase emerged that a starting point to cope with the above-mentioned issues could be trying to improve information and communication: that’s why the idea of a web portal, fed by volunteers, began to emerge as a possibility to identify the concrete needs and requests from Ukrainian refugees, in order to give them an effective reply.

In the policy implementation & simulation phase a first HTML draft of the home page was designed and the structure of the web portal was defined, i.e. a Ukrainian language website containing useful information on the following topics:

1) School in Ukraine;

2) School in Italy;

3) Sport and free time;

4) Renewal of residence permit (institutional information on the timing, offices, contact persons, together with the advice from people who have already experienced the same bureaucratic process etc...);

5) Housing: where to find a flat to rent (starting with the information already present on this subject on the City of Turin website, but translated into Ukrainian);

6) Health protection;

7) Job orientation.

The website environment is under development by the DECIDO team (specifically the members of the Turin cluster), as well as an editorial group of people speaking Ukrainian language is in the process of being established: this group is going to write articles, take care of the translations and in general to check the contents before their publication in the new website by the DECIDO team.

Hopefully the new website will facilitate the work of volunteers who are already managing a regional call centre in the Ukrainian language helping refugees in solving often complicated bureaucratic problems: the portal developed within the DECIDO project could at least represent a very first information hub to help Ukrainian asylum seekers access basic services, without replacing already existing initiatives or without pretending to solve very specific problems that have to be dealt individually by the competent authorities.

### First interpretation of results

At the time of writing (December 2022) all the three scenarios of the Turin pilot experience are undergoing the policy implementation & simulation phase. Therefore, the results of the policy evaluation phase are not yet available: they will be completed in spring 2023 and will be fundamental to understand if a positive change really took place in the response policies to the three emergencies under consideration.

However, it is already possible to provide a first interpretation, as well as the first experimental conclusions: first of all, the co-creation method, as a whole, has brought a large number of stakeholders belonging to often distant worlds (public, private and non-profit sectors) to meet and discuss together about possible shared solutions to common problems. So far, it has been a very successful problem-solving exercise: it has led participants to reflect on three emergencies that are very different from each other, but unified by the need to tackle a macro-problem segmentally, i.e. by dividing it into smaller problems to be dealt one at a time.

Moreover, this pilot experience has been the demonstration that citizens, regardless of their level of competence, can become protagonists of a helping relationship, not limiting themselves to being passive recipients of an aid or rescue intervention: an excellent sign in a period - such the one we are living in - where a general climate of environmental, geopolitical, and social uncertainty requires everyone to be prepared to face new challenges.

During this first round of the Turin pilot, it has been demonstrated that technologies can effectively support co-creation processes, even if face-to-face meetings have an added value: the DECIDO Portal has represented, in fact, a useful repository and management platform alongside each session.

Finally the participants, despite coming from different organisations and sectors, were able to put forward different points of view, without ever reaching a critical degree of conflict, but rather knowing how to harmonise diversities as sources of enrichment and maintaining the discussion on an always constructive level. In short, a good example of how it is possible to generate effective solutions to complex problems even starting from a bottom-up approach.

## Discussion

Thanks to funding received by the EU the DECIDO consortium is experimenting an innovative approach to re-define policy based on evidence. That is possible due to several factors starting from data availability and in particular EOSC open data. The importance of open data was demonstrated during the co-creation sessions in project pilot sites. Furthermore EOSC provided even dedicated services to support co-creation activities.

A delicate strategy to engage specific users was in place and achieved a very good result in engaging several tens of citizens with a large spectrum of competences in the co-creation process. Deep discussion, that is one of the key components of a useful evidence based policy re-design, was largely experimented and results in reliable KPIs to evaluate drafted policy.

Finally it is a unique experience the possibility to open a warm discussion among people who are not so used to address policy from the same viewpoint. In doing that DECIDO also introduces an overall hat with the DECIDO portal that provides in the same time access to both services and data collected from several open access sources together with a dedicated platform which allows following of the entire policy co-creation flow.

The project will leave a pending issue related to moving policies from the co-creation and simulation steps to the implementation supported by decision makers. We hope that by the end of the project (Feb 2024), a proposed pathway is going to be released to help further research in this field.

## Ethics and consent

Ethical approval and consent were not required.

## Data Availability

To create a policy we used a lot of data coming from different data sources (e.g environmental data, statistical data, etc.). As output of the DECIDO project we are creating the DECIDO Data Catalogue, that will contain all the datasets used and useful to create new policies. The DECIDO Data Catalogue will be published as an Open Data Portal ( https://decido.eng.it/decido-portal/pages/datasets/datasets-ui, this link could change when the final version of DECIDO Portal is published).
