# Supplementary material for: Evidence-based policy lifecycle management: The H2020 DECIDO experience
Source: Open Res Eur. 2023 Jul 20;3:70. Originally published 2023 May 2. [Version 2] doi: 10.12688/openreseurope.15697.2 (PMC10979129; doi:10.12688/openreseurope.15697.2)
Supplement: Peer review history

# Reviewer report: Reviewer response for version 2

**DOI:** 10.21956/openreseurope.17619.r38643

**Published:** 2024-03-28

**Authors:** Tatchalerm Sudhipongpracha

**Affiliations:** 1 College of Interdisciplinary Studies, Thammasat University, Bangkok, Bangkok, Thailand

The article reports the results of using a citizen engagement methodology (to which the authors refer as "co-creation" exercises) to elicit citizen inputs on seven (7) complex public policy problems. These co-creation exercises were conducted using an online platform. Open data were purportedly employed in this study, but the authors did not explicitly state what those open data were. 

 Please find below my comment on the questions to which I responded "no" or "partly":

**(1) Is the work clearly and accurately presented and does it cite the current literature?** Literature on citizen engagement (mechanisms, political/societal benefits, caveats) needs to be discussed more in the next version of this manuscript. Also, could the authors explain more how the open data platform works and how the data were used in the deliberative processes?

**(2) Are all the source data underlying the results available to ensure full reproducibility? **The authors should at least offer tables or figures that summarize the data underlying the results of this study. 

**(3) Are the conclusions drawn adequately supported by the results? **I still do not know what this case study is all about after finishing this manuscript. Even the discussion section would probably not be adequate for those wishing to use this manuscript as a case study. What will be fruitful is a discussion of the caveats associated with the co-creation method that policymakers and practitioners from other parts of Europe and the world need to know. 

**(4) Is the case presented with sufficient detail to be useful for teaching or other practitioners? **If the authors address my (1)-(3) comments, this article would be an interesting case study for practitioners and students of public policy and administration.

Is the case presented with sufficient detail to be useful for teaching or other practitioners?

No

Is the work clearly and accurately presented and does it cite the current literature?

No

If applicable, is the statistical analysis and its interpretation appropriate?

Not applicable

Are all the source data underlying the results available to ensure full reproducibility?

No

Are the conclusions drawn adequately supported by the results?

No

Is the background of the case’s history and progression described in sufficient detail?

Yes

Reviewer Expertise:

Local Government, Public Health Policy and Management, Foresight

I confirm that I have read this submission and believe that I have an appropriate level of expertise to confirm that it is of an acceptable scientific standard, however I have significant reservations, as outlined above.

# Reviewer report: Reviewer response for version 1

**DOI:** 10.21956/openreseurope.16959.r31573

**Published:** 2023-06-01

**Authors:** Andrea Bonomi Savignon

**Affiliations:** 1 Assistant Professor in Public Management, University of Rome Tor Vergata, Rome, Italy

The article presents a highly significant set of experiences in co-creation as applied to the policy lifecycle, with specific reference to emergency/disaster recovery management. First, it describes the role of co-creation across the different phases of the policy process. It then describes the approach of the DECIDO project in terms of its structural and technological components. Finally, the authors narrate three pilot experiences in the city of Turin where DECIDO tools are being implemented in the areas of flood emergencies, food waste, and asylum seekers management.

 I am glad to offer a number of comments that can help the authors refine the work and make it even clearer for the reader.

1. I think the abstract could be reviewed in order to make it clearer and more focused on the actual content of the paper. One way to do this is focusing it more on the concepts of co-creation and the use of digital tools for interactive policy making, and anticipating the contents of the three experiences presented. I do not see the ‘outcome of qualitative interviews’, cited here, as present in the actual text of the paper. In this light, the plain language summary is probably more consistent with the content of the paper and provides a better introduction to the reader.
2. The structure of the paper could be streamlined. A ‘Background’ section would be useful in order to introduce the core concepts of policymaking and co-creation, before the ‘Methods’ section. The ‘Methods’ section is somewhat misleading as it introduces the methods of the project and not of this particular paper. Earlier in the text, it could be useful to the reader to gather a better understanding of what co-creation is in public management literature, where it has been framed as a particular form of co-production when it happens at the ideation/governance phase of a service or policy; see for instance the paper by Nabatchi et al (2017) in the references ^1^. A quick reference to the citizen participation literature can also be useful (see for instance Fung, 2006, or Edelenbos & Klijn, 2005, also in the references here) ^2^^,^^3^.
3. The main area of improvement in the paper so far is that the cases (or pilot experiences) presented are limited to the context and results obtained, yet very little is described in terms of process. For instance, how many stakeholders were involved? How were they selected? How was representativeness ensured? How did they actually use and interact with the tools and technologies described earlier in the paper? These are key aspects of the co-creation initiatives that could also ensure replicability and scalability in terms of lessons learned.
4. Minor language revisions are needed throughout the text. For instance ‘solidarity unstructured self activated mechanisms’; ‘social value was an important driver for many initiatives to support fragile citizens than suddenly included all the city population’ (page 3). Also on page 3, you state ‘More than 18,000 families were assisted in an incredible self-organised way. That was an ante-litteram co-creation experience’. This should be carefully revised as self-organization and co-creation are two very different concepts.

I hope these comments can be useful to the authors in enhancing the potential of this very interesting paper (and project).

Is the case presented with sufficient detail to be useful for teaching or other practitioners?

Partly

Is the work clearly and accurately presented and does it cite the current literature?

Partly

If applicable, is the statistical analysis and its interpretation appropriate?

Not applicable

Are all the source data underlying the results available to ensure full reproducibility?

Partly

Are the conclusions drawn adequately supported by the results?

Partly

Is the background of the case’s history and progression described in sufficient detail?

Yes

Reviewer Expertise:

Public management; Co-production; Qualitative methods

I confirm that I have read this submission and believe that I have an appropriate level of expertise to confirm that it is of an acceptable scientific standard, however I have significant reservations, as outlined above.

## References

[1] : Varieties of Participation in Public Services: The Who, When, and What of Coproduction. *Public Administration Review* .2017;77(5) : 10.1111/puar.12765 766-776 10.1111/puar.12765

[2] : Varieties of Participation in Complex Governance. *Public Administration Review* .2006;66(s1) : 10.1111/j.1540-6210.2006.00667.x 66-75 10.1111/j.1540-6210.2006.00667.x

[3] : Managing Stakeholder Involvement in Decision Making: A Comparative Analysis of Six Interactive Processes in the Netherlands. *Journal of Public Administration Research and Theory* .2006;16(3) : 10.1093/jopart/mui049 417-446 10.1093/jopart/mui049

# Reviewer report: Reviewer response for version 1

**DOI:** 10.21956/openreseurope.16959.r31397

**Published:** 2023-05-05

**Authors:** Tsotne Zhghenti

**Affiliations:** 1 Business and Technology University, Tbilisi, Tbilisi, Georgia

This paper discusses the challenges faced by the DECIDO project in managing policy lifecycles, collecting and managing data and piloting suggested solutions to address citizens' needs and political representation issues in Europe. Therefore, the paper highlights concept of co-creation the importance of collecting and managing data.

 The COVID-19 pandemic and the Russian war in Ukraine are mentioned as examples of how citizens have shown solidarity in addressing emergencies using digital technologies. The pilot experience in Turin has demonstrated that citizens can become active participants in the policy development process and that technology can effectively support co-creation processes.

 The background of the case’s history is provided in the introduction part and later it is supported by relevant academic sources. Authors are also providing the preliminary findings as the project is ending in 2024. Therefore, the findings are only based on the first interpretation and not from the results. However, there is potential that final results would be a relevant material for teaching or other practitioners.

Is the case presented with sufficient detail to be useful for teaching or other practitioners?

Partly

Is the work clearly and accurately presented and does it cite the current literature?

Yes

If applicable, is the statistical analysis and its interpretation appropriate?

Not applicable

Are all the source data underlying the results available to ensure full reproducibility?

Yes

Are the conclusions drawn adequately supported by the results?

Yes

Is the background of the case’s history and progression described in sufficient detail?

Yes

Reviewer Expertise:

Digitalization, Economics.

I confirm that I have read this submission and believe that I have an appropriate level of expertise to confirm that it is of an acceptable scientific standard.
